# An Efficient Weed Detection Method Using Latent Diffusion Transformer for Enhanced Agricultural Image Analysis and Mobile Deployment

**DOI:** 10.3390/plants13223192

**Published:** 2024-11-13

**Authors:** Yuzhuo Cui, Yingqiu Yang, Yuqing Xia, Yan Li, Zhaoxi Feng, Shiya Liu, Guangqi Yuan, Chunli Lv

**Affiliations:** 1 College of Electrical and Information Engineering, China Agricultural University, Beijing 100083, China; 2 School of English and International Studies, Beijing Foreign Studies University, Beijing 100089, China

**Keywords:** weed detection, agricultural image analysis, latent diffusion transformer, real-time monitoring, deep learning

## Abstract

This paper presents an efficient weed detection method based on the latent diffusion transformer, aimed at enhancing the accuracy and applicability of agricultural image analysis. The experimental results demonstrate that the proposed model achieves a precision of 0.92, a recall of 0.89, an accuracy of 0.91, a mean average precision (mAP) of 0.91, and an F1 score of 0.90, indicating its outstanding performance in complex scenarios. Additionally, ablation experiments reveal that the latent-space-based diffusion subnetwork outperforms traditional models, such as the the residual diffusion network, which has a precision of only 0.75. By combining latent space feature extraction with self-attention mechanisms, the constructed lightweight model can respond quickly on mobile devices, showcasing the significant potential of deep learning technologies in agricultural applications. Future research will focus on data diversity and model interpretability to further enhance the model’s adaptability and user trust.

## 1. Introduction

In agricultural production, the management and control of weeds are crucial for ensuring healthy crop growth and increasing yields [1,2,3]. Weeds not only compete with crops for water, light, and nutrients but can also become potential hosts for pests and diseases, thereby increasing the risk of crop diseases [4,5,6]. Globally, weeds cause agricultural losses amounting to billions of dollars each year, which not only impact crop yields but also affect the quality of agricultural products. Therefore, the efficient and accurate identification and management of weeds are particularly important for modern agriculture. However, traditional weed management methods mostly rely on manual identification and the use of chemical herbicides, which are labor-intensive as well as inefficient and pose serious environmental risks due to the long-term extensive use of chemicals [7,8,9].

In contrast, the introduction of deep learning (DL)-based methods offers significant advantages in terms of accuracy, efficiency, and sustainability. While traditional manual inspections may work well with experienced field researchers, the scalability and speed of DL methods make them far more suitable for large-scale operations. With the development of precision agriculture, the use of image recognition and machine learning techniques for weed identification has gradually become a research hotspot [10]. Traditional machine learning methods have achieved some success in image processing, for example, using algorithms like support vector machine (SVM) [11] and random forest [12] for feature classification. However, these methods struggle when confronted with the variability in environmental factors, such as lighting, weather conditions, or image quality, leading to a reduction in reliability. For instance, Bhagat Monu et al. [13] used a SVM to classify plant leaves as healthy or unhealthy; for detecting plant leaf diseases, the recognition accuracy was only 80%. This highlights the need for more robust, automated systems that can provide real-time, precise analysis regardless of the challenging field conditions. Additionally, most of these algorithms require manual feature extraction, limiting the model’s generalizability and scalability.

In recent years, deep learning (DL) technologies, particularly convolutional neural networks (CNNs) and transformer models, have demonstrated exceptional performance in image recognition [14,15]. These models can automatically extract and process image features, significantly enhancing the accuracy and efficiency of crop recognition. Parez Sana et al. [16] proposed a fine-tuning technique called GreenViT based on Vision transformer (ViT) to detect plant infections and diseases. Similar to word embeddings, they divided input images into smaller blocks and sequentially fed them into the ViT. Their method leveraged the advantages of ViTs to overcome the issues associated with CNN-based models. Experiments conducted on widely used benchmark datasets showed that the proposed GreenViT outperformed state-of-the-art CNN models in detecting plant diseases; Biswas Srabani et al. [17] introduced a new energy-efficient CNN architecture and compared it with two existing models, VGG19 and Inception V3, achieving an accuracy of 95.17%. Ulutaş Hasan et al. [18] classified nine types of tomato plant leaf diseases and healthy leaves using DL and a new ensemble architecture, employing a total of 18,160 images. In this study, in addition the two newly proposed CNN models, four other well-known CNN models (MobileNetV3Small, EfficientNetV2L, InceptionV3, and MobileNetV2) were utilized. Fine-tuning methods were applied to the newly proposed CNN models, followed by hyperparameter optimization using particle swarm optimization (PSO). The experimental results indicated that the proposed ensemble model exhibited rapid training and testing times alongside excellent classification performance, achieving an accuracy of 99.60%. However, the computational resources required for model operation were often substantial, making it unsuitable for deployment on resource-constrained mobile devices.

Barman Utpal et al. [19] used a ViT model to identify healthy and unhealthy plants affected by diseases. A collected tomato leaf dataset was used to collectively train DL models based on Vision transformer and Inception V3 to distinguish between healthy and diseased plants. Chen et al. [20] proposed a transformer model based on cycle-consistent generative adversarial networks for generating images of diseased tomato leaves for data augmentation. Additionally, they used a transformer model and a densely connected CNN architecture to extract multilevel local features. The experiments showed that the proposed model achieved an accuracy of 99.45% on the PlantVillage dataset. However, the robustness of the model still needs further validation to adapt to complex and variable field environments.

In contrast, while much of the existing research has focused on using deep learning for image recognition to detect weeds, many of these methods have limitations, such as weak performance on low-quality images or low accuracy when facing complex backgrounds. Against this backdrop, this paper proposes a weed detection method based on a latent diffusion transformer to address the aforementioned issues. This method combines the advantages of latent variable models and diffusion models, enhancing the model’s robustness in processing complex, low-quality images by introducing a diffusion process in latent space; simultaneously, it improves identification accuracy by deeply learning and utilizing the deep features of images through transformer networks. In particular, when dealing with low-quality images, such as those with insufficient lighting or complex backgrounds, the model demonstrates stronger robustness and stability through the expansion of latent space and the denoising process. The main innovations of this research are as follows:

- A novel latent-based diffusion subnetwork employs a unique diffusion process in the latent space, effectively enhancing the model’s ability to handle complex and blurry image data. In agricultural applications, field-captured images are often affected by external factors such as lighting and weather, leading to variations in image quality. The latent-based diffusion subnetwork simulates the generation process of these images, enabling the reconstruction of or enhancement in key features even when image quality is poor, ensuring applicability and robustness in actual agricultural environments.
- The latent-based transformer subnetwork utilizes the self-attention mechanism of the transformer architecture to effectively extract key features from fine-grained images. This subnetwork is particularly suited for handling spatial relationships and complex patterns in images, such as the identification of different types of weeds. By deeply learning diverse features and patterns within the images, this network not only improves the accuracy of the recognition process but also enhances the model’s reliability in varying agricultural environments.
- An innovative latent loss function is introduced to optimize the training process and improve model performance. This loss function combines traditional loss functions with the specific needs of the model, optimizing the feature fusion and regression processes. The latent loss function precisely adjusts the model’s performance in latent space, allowing the network to focus more on areas with significant errors, thereby improving the model’s performance overall.

Through these innovations, this research not only provides a new perspective for utilizing DL technology in agricultural image analysis but also demonstrates significant potential and value in practical applications.

## 2. Related Work

### 2.1. Diffusion

Diffusion models, conceptualized from the physical diffusion process where substances move naturally from regions of high concentration to lower concentration, serve as generative models [21,22,23]. In the realm of machine learning, these models articulate the transition from a purely random distribution, typically Gaussian noise, to a structured data distribution [24,25,26]. The essence of diffusion models lies in defining a process of gradual denoising that simulates the reverse generation of data [27,28,29]. Comprising two primary phases—the forward diffusion process that introduces noise and the reverse generation process that eliminates it—these models encapsulate a comprehensive approach to data synthesis.

During the forward diffusion phase, noise is systematically introduced into the data until they are entirely converted into noise. This process is described by the following equation:

(1) $$q\left(x_{t}|x_{t-1}\right)=N\left(x_{t};\sqrt{1-\beta_{t}}x_{t-1},\beta_{t}I\right)$$

where $x_{t}$ represents the data state at time step *t*, $\beta_{t}$ denotes the predetermined level of noise, N symbolizes a Gaussian distribution, and I is the identity matrix. The reverse generation process, conversely, is the antithesis of the forward process, where the model progressively recovers the original data from pure noise. The focus of this phase is on predicting the denoised data, which can be expressed through the following conditional probability distribution:

(2) $$p_{\theta }\left(x_{t-1}|x_{t}\right)=N\left(x_{t-1};\mu_{\theta }\left(x_{t},t\right),\sigma_{t}^{2}I\right)$$

In this formulation, $\mu_{\theta }\left(x_{t},t\right)$ is the mean function determined by the model parameter θ, and $\sigma_{t}^{2}$ represents the noise variance. In practical applications, diffusion models iteratively optimize the θ parameters, thereby gradually aligning the synthesized data with the actual data distribution. These models excel in processing image data and are capable of refining image details and textures across various scales, thus facilitating the production of high-quality images. Within the context of this research, diffusion models were employed to enhance the quality of weed images, particularly those captured under adverse environmental conditions. For instance, the quality of images taken during rainy days or under low-lighting conditions often suffers; here, diffusion models can leverage their robust generative capabilities to reconstruct or enhance critical information within the images [30,31]. This enhancement is vital for subsequent weed recognition and detection, as accurate image recognition relies on the integrity and clarity of the information within the images.

Moreover, by integrating the self-attention mechanism of transformer models, the accuracy and efficiency of recognition are further improved [32,33]. Transformer models, focusing on key areas within the image, such as specific types of weeds, optimize the flow and processing of information, thereby refining the recognition process at a granular level [34,35]. The synergistic use of diffusion models and transformer models not only boosts performance in weed detection tasks but also introduces new research directions and possibilities within the agricultural image processing field.

### 2.2. Transformer

Since its introduction by Google in 2017, the transformer model has become a revolutionary technology for processing sequential data, particularly in the field of natural language processing (NLP) [36]. Its core mechanism, the self-attention mechanism, is capable of capturing long-distance dependencies within sequences, which is crucial for understanding and generating language. Subsequently, applications of transformer have rapidly expanded to other areas, including computer vision tasks such as image classification, object detection, and image segmentation [37,38,39].

The primary components of the transformer model include the multihead self-attention mechanism, positional encoding, and feed-forward neural networks [40]. These components collaborate to process and generate complex patterns in sequence data. The self-attention mechanism calculates an attention score for each element in the sequence, determining the importance of other elements when forming a representation of the current element. In image processing, each area of the image, such as pixels or image blocks, can be considered an element in sequence processing. The self-attention is calculated as follows:

(3) $$\mathrm{Attention}\left(Q,K,V\right)=\mathrm{softmax}\left(\frac{QK^{T}}{\sqrt{d_{k}}}\right)V$$

Here, *Q*, *K*, and *V* represent the query, key, and value matrices, respectively, derived from input data through different weight matrices. $d_{k}$ is the dimension of the key vectors, which serves as a normalization factor to prevent gradient-vanishing issues. The transformer model incorporates multihead self-attention to allow parallel learning of different features of the input data across various representational subspaces:

(4) $$\mathrm{MultiHead}\left(Q,K,V\right)=\mathrm{Concat}\left({\mathrm{head}}_{1},\ldots ,{\mathrm{head}}_{h}\right)W^{O}$$

(5) $${\mathrm{head}}_{i}=\mathrm{Attention}\left(QW_{i}^{Q},KW_{i}^{K},VW_{i}^{V}\right)$$

where $W_{i}^{Q}$, $W_{i}^{K}$, $W_{i}^{V}$, and $W^{O}$ are trainable weight matrices, and *h* denotes the number of heads.

Since the transformer model inherently lacks the capability to process the order of the sequences, positional encoding is introduced to provide the model with information about the position of the elements within the sequence. Positional encoding typically involves adding a fixed pattern to the embeddings of input elements:

(6) $$PE\left(pos,2i\right)=\sin\left(pos/{10,000}^{2i/d_{\mathrm{model}}}\right)$$

(7) $$PE\left(pos,2i+1\right)=\cos\left(pos/{10,000}^{2i/d_{\mathrm{model}}}\right)$$

where pos is the position of the element in the sequence, *i* is the dimension index, and $d_{\mathrm{model}}$ is the dimension of the embedding.

In the domain of vision, the transformer model effectively processes the spatial relationships within images, thereby achieving higher accuracy in recognizing multiple objects in complex scenes [41]. For example, ViT [42] represents a typical application of transformer in image classification, where images are segmented into multiple small pieces (patches), which are then processed through a standard transformer structure.

In agricultural image processing applications, transformer models effectively identify and classify different types of crops and weeds, especially in scenes with complex backgrounds or a diverse array of crops and weeds [43,44]. Additionally, by integrating positional encoding, transformer models can more accurately locate weed areas within images, providing a scientific basis for precise treatment and management [45,46]. In the context of this study, the transformer model was incorporated to enhance the capability to recognize weed features within agricultural images. Utilizing the self-attention mechanism, the model not only captures local features within the image but also understands their relative importance across the entire image, which is particularly crucial for accurately identifying weeds against complex backgrounds.

## 3. Materials and Method

### 3.1. Dataset Collection

**Figure 1:**
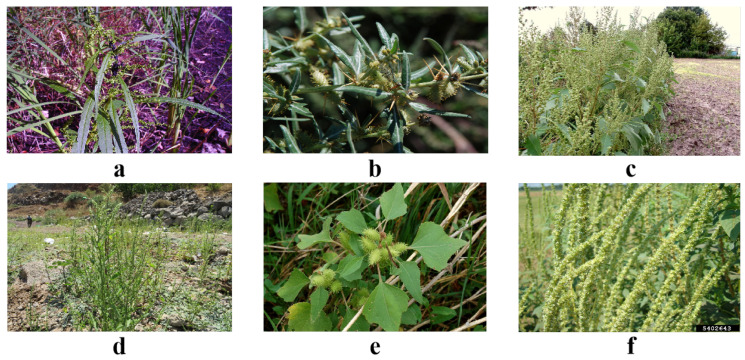
Dataset samples. (**a**) *Amaranthus retroflexus*, (**b**) *Xanthium spinosum*, (**c**) *Xanthium sibiricum*, (**d**) *Amaranthus palmeri*, (**e**) *Xanthium italicum*, (**f**) *Amaranthus hybridus*.

In this study, a dataset was constructed for weed detection, covering six major weed species: *Amaranthus retroflexus* (redroot pigweed), *Xanthium spinosum* (spiny cocklebur), *Xanthium sibiricum* (common cocklebur), *Amaranthus palmeri* (Palmer amaranth), *Xanthium italicum* (Italian cocklebur), and *Amaranthus hybridus* (hybrid amaranth), as shown in Figure 1.

These weeds are commonly found during agricultural production and are significant competitors with crops. Therefore, accurately identifying these weeds is crucial for effective agricultural management and yield protection. The dataset also includes images where multiple weed species appear within a single frame, providing a more realistic representation of field conditions. The dataset includes 800 to 1700 images of each weed species, primarily collected in Beijing and Changchun, Jilin province, and some online resources were used to ensure the richness and representativeness of the samples. The number of images collected for each weed species is shown in Table 1.

During the data collection process, a detailed investigation of the biological characteristics of the target weeds was conducted to clarify the growth environment, morphological features, and distribution of each species. For example, *Amaranthus retroflexus* is an annual herb, typically growing between 30 and 100 cm in height, with ovate leaves that are dark green, and its inflorescences are spike-like, producing small, hard seeds that turn black upon maturity. *Xanthium spinosum* is a perennial herb with spiny stems that can reach up to 1 m, featuring large heart-shaped leaves with serrated edges, often covered with white hairs on the underside. *Xanthium sibiricum* is similar to *Xanthium spinosum* but has different fruit morphology. *Amaranthus palmeri* is a resilient weed that typically grows between 30 and 120 cm tall, with broad leaves and spike-like inflorescences, producing small black seeds. *Xanthium italicum* resembles *Xanthium spinosum* in appearance, is highly adaptable, and can thrive in various soil conditions, commonly found in fields and abandoned lands. *Amaranthus hybridus* is an annual plant with upright stems that can grow up to 1 m tall, featuring long, oval-shaped leaves that vary in color from green to purple, with dense spike-like inflorescences and small seeds.

In the implementation phase of data collection, rigorous scientific methods were followed to ensure data accuracy and reliability. Choosing an appropriate time for collection was critical; capturing images primarily in the early morning and late evening helped avoid issues with the shadows and highlights caused by intense mid-day sunlight, thus enhancing overall image quality. Additionally, weather conditions were carefully monitored, prioritizing sunny or overcast days for photography while avoiding rain and strong winds, which could affect image quality. To ensure the collected images had good clarity and recognizability, a high-resolution Canon EOS 90D DSLR camera (Tokyo, Japan) was used, along with lenses of varying focal lengths (including 18–55 mm and 50 mm), effectively capturing the detailed features of the weeds. In terms of specific collection methods, suitable locations for sampling were identified, focusing on areas where the target weeds were abundantly present, such as farmlands, roadsides, and some relatively hidden grass patches. The selected collection points ensured sample diversity and representativeness, with multiple species often photographed together in the same frame. Thus, multiple sampling points were randomly chosen at each site. At each sampling point, efforts were made to photograph the same plant from various angles, including front, side, and bottom views, to obtain comprehensive samples. During the image collection process, tripods were used to ensure shooting stability. This measure not only effectively prevented blurriness caused by handshaking but also maintained consistent height and angle during photography.

To control image quality, a strict review mechanism was established. After each collection session, the quality of the captured images was promptly assessed, removing any blurry, overexposed, or underexposed images to ensure the high quality of the final dataset. Images with overexposure or underexposure issues were automatically removed using traditional visual methods, such as white balance adjustments. Beyond visual quality, each image was annotated with information including weed species, collection location, collection time, and growth environment. This information not only provided rich context for subsequent model training but also aided the researchers in better understanding the data background during result analysis. Through this systematic approach to collection and annotation, over 8000 weed images were gathered, encompassing the range of these six weed species. These images not only provide essential foundational data for model training but also lay a solid groundwork for future experimental design and result analysis. Moreover, special attention was paid to data annotation and management during the dataset construction process, ensuring that each sample was accurately classified to enhance the efficiency and effectiveness of model training.

### 3.2. Image Augmentation

A series of sophisticated and effective image augmentation techniques were employed to enhance the robustness and adaptability of the model in weed detection tasks. Aimed at increasing the model’s generalization capabilities in varying environments, these techniques specifically addressed the image quality issues arising from natural factors such as lighting and weather. The dataset images were collected with high resolution, specifically 1920 × 1080 pixels, which ensured that the details in the images were fully displayed. This resolution is especially crucial when processing fine features of plants, such as leaf shape, size, and disease manifestations, as it provides higher clarity for these characteristics. High-resolution images offer more pixel information, which was essential for the model to learn subtle differences in the images, improving its ability to differentiate between weed species and identify diseases. The aspect ratio of the images was determined based on the real-life growth patterns of the weeds, ensuring that most images had an aspect ratio close to 4:3 or 16:9, which is consistent with standard agricultural image capture guidelines and ensures diversity and representativeness in a dataset.

**Figure 2:**
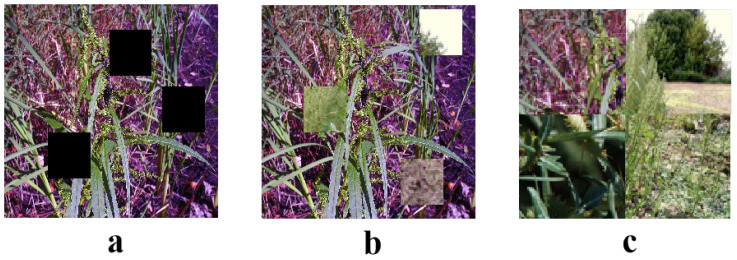
Dataset augmentation. (**a**) CutOut, (**b**) CutMix, (**c**) Mosaic.

In the preprocessing steps, normalization and magnification techniques were primarily applied. For each image, pixel values were normalized to the range of [0, 1], ensuring uniform brightness and contrast across all images. This helped to minimize the variation caused by different lighting conditions and prevented issues like uneven lighting or over/under-exposure from affecting the training process. Normalization also accelerated the model’s convergence speed, as it ensured the input data were within a certain range, preventing any single feature from dominating the training process, which improved the stability of the gradient descent method. In addition to these traditional enhancements, advanced data augmentation techniques, such as CutOut, CutMix, and Mosaic, were used, as shown in Figure 2. These methods helped simulate real-world scenarios where weeds might appear in various contexts, enhancing the model’s robustness and adaptability. These techniques were employed to further address image quality issues arising from natural conditions, such as variations in lighting and background clutter, making the model more resilient to these real-world challenges.

CutOut, a simple yet effective data augmentation technique, enhances a model’s ability to recognize occluded objects by randomly masking parts of the image. In particular, the CutOut operation involves randomly selecting a rectangular area in the training image and setting the pixel values within this area to zero or the image’s mean value. This method simulates real-world visual scenarios where partial occlusion might occur, such as parts of vegetation being obscured by other objects or plants. The CutOut operation is defined by the following equation:

(8) $$I^{\prime }\left(x,y\right)=\left\{\begin{array}{cc} 0 & \mathrm{if}\;\left(x,y\right)\in \mathrm{Rect}\left(x_{c},y_{c},w,h\right)\\ I(x,y) & \mathrm{otherwise} \end{array}\right.$$

where I(x,y) represents the pixel value in the original image, and $\left(x_{c},y_{c}\right)$, *w*, and *h* are the center coordinates, width, and height of the rectangle, respectively. CutMix extends this concept further by not only masking an area in one image but also filling the masked area with a corresponding part from another image. This technique not only enhances the model’s adaptability to occlusions but also improves its capability to handle diverse image information. Mathematically, CutMix can be described as

(9) $$I^{\prime }\left(x,y\right)=\left\{\begin{array}{cc} I_{2}\left(x-x_{c}+x_{c2},y-y_{c}+y_{c2}\right) & \mathrm{if}\;\left(x,y\right)\in \mathrm{Rect}\left(x_{c},y_{c},w,h\right)\\ I_{1}\left(x,y\right) & \mathrm{otherwise} \end{array}\right.$$

where $I_{1}$ and $I_{2}$ are the two images selected for mixing, and $\left(x_{c2},y_{c2}\right)$ is the center of the cropped area in the second image. Mosaic augmentation involves stitching together four different images into one new image. This method not only enhances the model’s ability to recognize local features in images but also significantly expands the diversity of visual scenes. The Mosaic operation is defined as follows:

(10) $$I^{\prime }\left(x,y\right)=I_{i}\left(\mathrm{mod}\left(x,w/2\right),\mathrm{mod}\left(y,h/2\right)\right)\;\mathrm{for}\;i=1,2,3,4$$

where *w* and *h* are the width and height of the target image, and $I_{i}$ is the image part taken from the *i*-th image used in the stitching. The application of these image augmentation techniques significantly enhanced the complexity and diversity of the dataset, laying a solid foundation for training a robust weed detection model. These techniques not only simulated the various visual challenges encountered in agricultural scenarios, such as partial occlusions and mixing of different vegetation types, but also enhanced the model’s adaptability and recognition capabilities in these complex situations.

### 3.3. Proposed Method

#### 3.3.1. Overall

**Figure 3:**
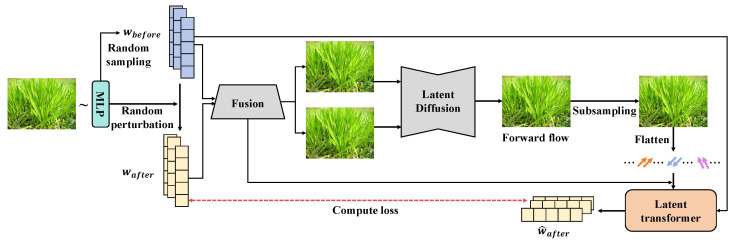
The overall structure diagram of the weed disease detection method based on a latent diffusion transformer. This figure illustrates the various modules of the model, including the latent-based diffusion subnetwork, latent-based transformer subnetwork, and latent loss function, reflecting the complete process of data input, feature extraction, information processing, and output.

The weed detection method proposed in this study adopts a multimodule DL framework, with the overall process designed to enhance the processing and recognition capabilities of complex agricultural images through the integration of a latent diffusion transformer. This method is primarily divided into three core modules: the latent-based diffusion subnetwork, the latent-based transformer subnetwork, and the latent loss function, as shown in Figure 3. Each module plays a specific role in the overall workflow, achieving gradual optimization of data and feature extraction through efficient connections.

First, the preprocessed image data are input into the latent-based diffusion subnetwork. In this module, the image data are transformed into a latent space representation. By defining a diffusion process that gradually introduces and removes noise, the model can reconstruct and enhance key features when processing low-quality or blurred images. The mathematical expression for the diffusion process is as follows:

(11) $$x_{t}=\sqrt{\alpha_{t}}x_{0}+\sqrt{1-\alpha_{t}}\epsilon$$

where $x_{t}$ represents the current state of the image, $x_{0}$ is the original input, ϵ is the random noise, and $\alpha_{t}$ is the parameter that controls the noise. After processing through multiple diffusion layers, the image features gradually become clearer, laying a solid foundation for subsequent recognition tasks. Next, the output latent space features are passed to the latent-based transformer subnetwork. In this module, the model further analyzes and understands the extracted features through the self-attention mechanism. The calculation of the self-attention mechanism allows the network to dynamically focus on important features, capturing subtle changes and details in the image. This process not only enhances the expressiveness of the features but also ensures effective information flow, allowing the model to exhibit higher accuracy when processing diverse images. Finally, the output of the latent-based transformer subnetwork is passed through a fully connected layer to generate the final feature representation, which is combined with the latent loss function. In this phase, the loss function is optimized based on the model’s predictions and the true labels, ensuring that the model can focus on areas with significant errors. This optimization process not only improves training efficiency but also enhances the model’s generalization capability. The design of the latent loss function ensures that the model can continuously adjust during the training process, further improving various performance metrics. Through the efficient interconnection of these modules, the proposed weed detection method performs a complete process from data input to feature output. The interaction between modules ensures the flow and optimization of information, providing the model with robust recognition capabilities and adaptability in practical applications and offering a new solution for agricultural image analysis. Overall, the design logic of this method is clear, and the modular division is reasonable, laying a solid foundation for future research and applications.

#### 3.3.2. Latent-Based Diffusion Subnetwork

**Figure 4:**
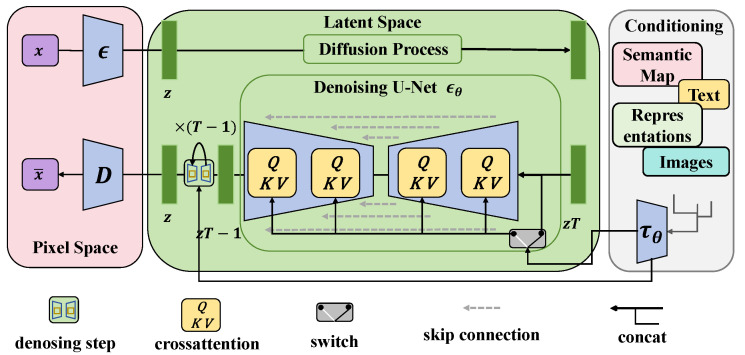
Schematic diagram of the latent-based diffusion subnetwork. This figure presents the architecture of the latent-based diffusion subnetwork, which includes the input layer, multiple diffusion layers, and the output layer. It highlights the process of progressive denoising and feature reconstruction to effectively enhance the feature extraction capability of complex agricultural images.

The latent-based diffusion subnetwork is one of the core innovations of this study, designed to effectively handle complex and low-quality image data, particularly for the detection of weeds in agricultural scenarios. The design of this subnetwork is primarily based on the fundamental principles of diffusion models, which restore and enhance the key features of the input image through a gradual denoising process, as shown in Figure 4. The specific implementation and design parameters of this subnetwork are detailed below.

In terms of network architecture, the latent-based diffusion subnetwork consists of multiple layers, adopting a progressive structure for feature extraction and enhancement. The specific design includes an input layer, several diffusion layers, and an output layer. The size of the input layer was set to 128×128×3, corresponding to the width, height, and number of channels of the input image, where 3 represents the RGB color channels. This size selection effectively captures the basic features in the image while ensuring computational efficiency in the subsequent processing. In the design of the diffusion layers, this subnetwork employs five diffusion layers, each with a width and height of 64×64 and a channel count of 64. Convolution operations are utilized in each diffusion layer for feature extraction, employing a 3×3 convolution kernel for local feature learning. The convolution operation can be expressed as follows:

(12) $$Y\left[i,j\right]=\sum_{m=-1}^{1}\sum_{n=-1}^{1}X\left[i+m,j+n\right]\cdot K\left[m+1,n+1\right]$$

where Y[i,j] represents the value of the output feature map, *X* is the input feature map, and *K* is the convolution kernel. After each diffusion layer, batch normalization and ReLU activation functions are introduced to enhance the network’s nonlinear expression ability and accelerate the training process. In the final output layer, a 1×1 convolution kernel is used to compress the channel count of the feature map to the final target number, which is the output feature dimension. The size of the output layer is related to the required final feature dimension, typically set to 32 channels, effectively retaining key information from the image and facilitating processing by the subsequent transformer subnetwork. The overall architecture of the latent-based diffusion subnetwork can be summarized as follows:

- Input layer: 128×128×3;
- Diffusion layers 1–5: each layer 64×64×64;
- Output layer: 64×64×32.

Through this network structure, features at different levels can be progressively extracted and enhanced, ultimately forming high-quality feature maps suitable for processing by the transformer. From a mathematical perspective, the key to the diffusion process lies in the gradual introduction and removal of noise. Specifically, the diffusion process can be described using the following formula:

(13) $$x_{t}=\sqrt{\alpha_{t}}x_{0}+\sqrt{1-\alpha_{t}}\epsilon$$

where $x_{t}$ represents the image state at time *t*, $x_{0}$ is the original image, ϵ is Gaussian noise, and $\alpha_{t}$ is the hyperparameter controlling the level of noise introduction. During training, the parameter $\alpha_{t}$ is continuously adjusted to achieve the denoising and feature enhancement of the image. This design has significant advantages when processing agricultural images. First, in addressing the issue of varying image quality due to environmental changes, the latent-based diffusion subnetwork can effectively reconstruct or enhance key features by simulating the image generation process, ensuring the model’s applicability and robustness in real agricultural environments. Second, the subnetwork processes features progressively at multiple levels, allowing the network to learn richer and more complex feature representations, thereby improving the accuracy of the subsequent recognition tasks.

#### 3.3.3. Latent-Based Transformer Subnetwork

**Figure 5:**
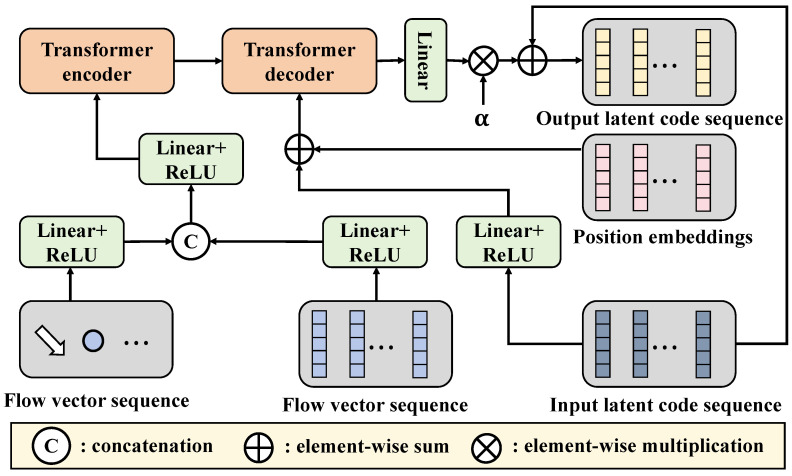
Schematic diagram of the latent-based transformer. This figure showcases the structure of the latent-based transformer, including the self-attention mechanism, multiscale feature extraction, and feed-forward neural network layers. It emphasizes how features are processed and fused in the latent space to enhance the accuracy and stability of weed disease detection.

The latent-based transformer subnetwork is an important component of this method, aimed at enhancing the feature extraction and understanding the capabilities with complex images in the weed detection task. This subnetwork not only inherits the advantages of traditional transformer models but also strengthens the model’s ability to capture image details and contextual information by introducing latent space representations, as shown in Figure 5.

Traditional transformer models are primarily used for processing sequential data, utilizing the self-attention mechanism to capture the relationships between elements in the input sequence. The core idea of the self-attention mechanism is to dynamically adjust the information flow by calculating the similarity between input elements. However, traditional transformers often face high computational costs and insufficient detail capture when dealing with high-dimensional image data. To address this issue, the following design adjustments were made to the latent-based transformer subnetwork:

1. Latent space representation: This subnetwork transforms the input images into latent space representations, processing them through the output of the latent-based diffusion subnetwork. This representation allows for the extraction of more abstract features, reducing redundant information.
2. Multiscale self-attention: In the latent-based transformer, a multiscale self-attention mechanism is introduced, which enables the learning of image features at different scales and increases the focus on details.
3. Extended input dimensions: By expanding the input dimensions to multiple channels, the network can simultaneously pay attention to various types of features in the image, achieving more comprehensive information extraction.

The mathematical expression for the latent-based transformer subnetwork includes the self-attention mechanism and multiscale processing. The specific self-attention calculation formula is similar to that of the traditional transformer, but it uses the feature representations processed by the latent-based diffusion subnetwork. Building on this, multiscale feature representations are introduced. After processing through the self-attention layers, the feature maps enter the feed-forward neural network layers to further enhance the nonlinear expression capability of the features. The output of this layer can be represented as

(14) $$\mathrm{FFN}\left(x\right)=\sigma (W_{2}\cdot \mathrm{ReLU}\left(W_{1}\cdot x+b_{1}\right)+b_{2})$$

where $W_{1}$ and $W_{2}$ are learnable weight matrices, $b_{1}$ and $b_{2}$ are bias terms, and σ denotes the activation function. Through this design, the latent-based transformer subnetwork can effectively extract and fuse features at different levels, thereby improving the performance of the model. The advantages of the latent-based transformer subnetwork when processing complex images lie in its multiscale self-attention mechanism. By focusing on features at different scales, the network can better adapt to the deformations and complexities within images. Let the feature representation of an image be $X\in R^{H\times W\times C}$, where *H*, *W*, and *C* represent the height, width, and number of channels of the image, respectively. In multiscale processing, feature maps generate multiple feature representations $X_{i}\in R^{H_{i}\times W_{i}\times C_{i}}$ after passing through convolution and pooling operations at different scales. Through the self-attention mechanism, information from different scales can be effectively integrated:

(15) $$X_{\mathrm{final}}=\sum_{i}{\mathrm{Attention}}_{i}\left(Q_{i},K_{i},V_{i}\right)$$

This weighted summation method ensures that important features can be better recognized and utilized, improving the accuracy of detection. The design of the latent-based transformer subnetwork offers multiple advantages, particularly in agricultural image processing applications. First, by introducing latent space, the model can more effectively handle the noise and blurriness in images, maintaining high recognition accuracy even under low-quality conditions. Second, the multiscale self-attention mechanism allows the network to adapt to changing backgrounds and enhance detail capture. This capability is particularly important for recognizing various types of weeds, as these features often exhibit significant variability in images. Furthermore, the design of this subnetwork enhances the model’s generalization ability. During training, the model not only learns features from specific samples but also comprehends the overall feature distribution. This ensures that the model can effectively adapt to new environments and conditions when processing diverse images in real agricultural settings.

#### 3.3.4. Latent Loss Function

The latent loss function is a key innovation in this study, aimed at optimizing the model training process and improving the performance of the model in weed detection tasks. Compared to traditional loss functions, the latent loss function enhances model performance and stability by specifically optimizing the features in latent space. Traditional loss functions, such as Mean Squared Error (MSE) and cross-entropy loss, primarily focus on directly assessing the difference between model predictions and true labels. While these loss functions perform well in many tasks, they may fail to adequately capture the multilevel features of data, especially in complex high-dimensional data and DL models. For instance, in image classification tasks, simple loss functions may not effectively handle the similarities and differences between different classes. In contrast, the latent loss function not only considers the error between model outputs and true values but also incorporates the specific requirements of the task, optimizing the fusion and regression of features. Specifically, the design of the latent loss function aims to focus on the model’s performance in latent space, utilizing importance weighting of features to better adjust the model’s focus when faced with complex data. The basic form of the latent loss function can be expressed as

(16) $$L=\lambda_{1}L_{\mathrm{reconstruction}}+\lambda_{2}L_{\mathrm{regularization}}+L_{\mathrm{specific}}$$

where $L_{\mathrm{reconstruction}}$ is the reconstruction loss, $L_{\mathrm{regularization}}$ is the regularization loss, $L_{\mathrm{specific}}$ is the task-specific loss term, and $\lambda_{1}$ and $\lambda_{2}$ are hyperparameters used to balance the effects of different loss components. The reconstruction loss $L_{\mathrm{reconstruction}}$ is specifically defined as

(17) $$L_{\mathrm{reconstruction}}=\frac{1}{N}\sum_{i=1}^{N}\left|\right|{\hat{y}}_{i}-y_{i}{\left|\right|}^{2}$$

where ${\hat{y}}_{i}$ is the model’s predicted output, $y_{i}$ is the true label, and *N* is the number of samples. This loss measures the mean squared error between the model’s output and the true values, ensuring accuracy in reconstructing features. The regularization loss $L_{\mathrm{regularization}}$ typically adopts an L2 regularization form:

(18) $$L_{\mathrm{regularization}}=\frac{\lambda_{r}}{2}\sum_{j=1}^{M}\left|\right|W_{j}{\left|\right|}^{2}$$

where $W_{j}$ is the weight of the *j*-th layer in the model, *M* is the number of layers, and $\lambda_{r}$ is the regularization strength. By introducing the regularization term, the model can prevent overfitting and improve its generalization ability on unseen data. The specific loss term $L_{\mathrm{specific}}$ can be designed based on task-specific requirements, such as weighting for certain classes:

(19) $$L_{\mathrm{specific}}=\sum_{k=1}^{C}w_{k}\cdot L_{k}$$

where $w_{k}$ is the weight for class *k*, $L_{k}$ is the loss for class *k*, and *C* is the total number of classes. This design allows the model to appropriately adjust its focus when facing different classes, enhancing sensitivity to rare or important categories. To demonstrate the effectiveness of the latent loss function, consider the model’s performance during the training process. Let the output of a simple model be defined as $\hat{y}=f\left(x;W\right)$, where *x* is the input data, and *W* is the model parameters. According to the definition of the loss function, the objective is to minimize the loss:

(20) $$\min_{W}L\left(W\right)=\min_{W}\left(\lambda_{1}L_{\mathrm{reconstruction}}+\lambda_{2}L_{\mathrm{regularization}}+L_{\mathrm{specific}}\right)$$

By updating the *W* parameters using gradient descent, we can obtain

(21) $$W^{\left(t+1\right)}=W^{\left(t\right)}-\eta \nabla L\left(W^{\left(t\right)}\right)$$

where η is the learning rate, and $\nabla L(W^{\left(t\right)})$ is the gradient at the current parameters. The latent loss function effectively adjusts the direction and magnitude of the gradients by combining different loss components, ensuring that the model focuses not only on reconstruction accuracy but also on the rationality and importance of the features during training. In the weed detection task, the latent loss function exhibits significant advantages. First, by emphasizing feature expression in latent space, this loss function allows the model to capture key features more effectively under low-quality image conditions. By optimizing reconstruction loss, the model can accurately recover crucial information in blurred images, ensuring good applicability in real agricultural environments. Second, the introduction of regularization loss effectively prevents overfitting and enhances the model’s generalization ability. The diversity and complexity of the data in agricultural scenarios require the model to adapt to different environments and conditions, which is precisely what the design of the latent loss function addresses. Finally, the use of specific loss terms allows the model to flexibly adjust to actual application needs.

### 3.4. Experimental Configuration

Experiments were conducted on servers equipped with high-performance GPUs. Specifically, NVIDIA Tesla V100 GPUs (Santa Clara, CA, USA), each with 32 GB of memory, were utilized, providing ample computational resources for processing large-scale agricultural image data and complex DL models. These high-performance GPUs facilitated faster data processing speeds and more efficient parallel computing capabilities, making them ideal for high-quality DL experiments. In terms of software, all experiments were performed under the Linux operating system, utilizing the Python programming language (version 3.8) and the PyTorch framework (version 1.8). PyTorch offers robust library support, including an automatic differentiation system (Autograd) and a comprehensive set of predefined DL modules, along with strong community support and a broad developer base. These features make it well suited for rapid prototyping and iterative experimentation.

The choice of hyperparameters plays a decisive role in the learning outcomes and generalization capabilities of DL models. In this study, the Adam optimizer, a method based on adaptive estimation, was employed to automatically adjust the learning rates of individual parameters, particularly effective for large-scale problems with nonuniform data updates. The initial learning rate was set to 0.001. Additionally, to optimize the learning process and prevent overfitting, a learning rate decay mechanism was introduced. Learning rate decay, a common technique, gradually reduces the learning rate during training to ensure more stable convergence as the model approaches the optimal solution. If no improvement in loss on the validation set was observed over ten consecutive epochs, the learning rate was reduced by a factor of 0.9, helping to prevent excessive oscillations in the loss landscape as the model neared its optimal state.

Batch size, a critical hyperparameter determining the number of data samples used for gradient computation in each iteration, was set to 32. This size was chosen considering the GPU memory capacity and training efficiency, ensuring sufficient data volume for reliable gradient calculation while also maximizing the use of GPU resources. The training was planned for 100 epochs, which was generally sufficient to ensure that most model parameters adequately learned from the training data. To further control overfitting, an early stopping strategy was implemented, where training was prematurely terminated if no improvement in performance on the validation set was observed over 20 consecutive epochs. This strategy not only conserved computational resources but also prevented the model from overfitting to the training data.

To comprehensively evaluate the model’s performance and generalization ability, 10 repetitions of 5-fold cross-validation (5-fold CV) were employed. Specifically, the dataset was divided into five nonoverlapping subsets, with four subsets used as training data and the remaining subset used for validation during each training iteration. In this study, 80% of the images were used for training, 10% for validation, and the remaining 10% for testing. Specifically, the number of images in the training set for each weed species ranged from 640 to 1360 images, while the number of images in the validation and testing sets ranged from 80 to 170 images. Each experimental method (including the proposed method) was repeated 10 times, with different training and validation sets chosen in each repetition. The results from the 10 repetitions were then averaged to provide a more stable and reliable evaluation. This method effectively utilized limited data resources and provided a more accurate estimation of the model’s performance on unseen data.

### 3.5. Baseline

In this study, several advanced object detection and segmentation models were selected as baselines to evaluate the effectiveness of the proposed method, specifically YOLOv9 [47], EfficientDet [48], TinySegformer [49], and DETR (detection transformer) [50]. The design philosophy of YOLOv9 focuses on achieving fast and efficient real-time object detection. YOLOv9 completes detection tasks through a single forward propagation process, avoiding the inefficiencies associated with multiple region proposals in traditional methods. The network architecture of YOLOv9 introduces depthwise separable convolutions and cross-layer connections to reduce computational load and enhance feature extraction capabilities. EfficientDet is an efficient object detection model based on the EfficientNet architecture. It employs a compound scaling method to effectively balance the model’s depth, width, and resolution, thereby reducing computational resource consumption while maintaining high accuracy. EfficientDet utilizes a bi-directional feature pyramid network (BiFPN) to efficiently fuse features of different scales. TinySegformer, on the other hand, is a lightweight image segmentation model specifically designed for embedded devices and mobile platforms. It combines the advantages of transformers with the efficiency of convolutional neural networks, utilizing a multihead self-attention mechanism for feature extraction. Lastly, DETR introduces transformers into the field of object detection, featuring end-to-end training capabilities. DETR models the long-range dependencies in images through a global self-attention mechanism, allowing for efficient parsing of complex scenes. Each of these baseline models offers unique strengths that, when combined with the latent diffusion transformer approach, provide a robust foundation for addressing the challenges associated with weeds and their detection in agricultural settings.

### 3.6. Evaluation Metrics

Multiple evaluation metrics were utilized to comprehensively assess the performance of the weed detection system, ensuring its efficiency and precision in practical applications. These metrics, including precision, recall, accuracy, mean average precision (mAP), and F1 score, reflected the model’s performance from various perspectives, aiding in the understanding and targeted optimization of the model. Precision, a critical indicator of predictive accuracy, measures the proportion of positive identifications that are correct. Recall, another key metric, assesses the proportion of actual positives that are correctly identified by the model. The significance of recall in weed detection is directly linked to the system’s risk of missing weeds. Accuracy, the most intuitive performance metric, indicates the proportion of samples that are correctly classified. Particularly in cases where the class distribution is balanced, accuracy provides a quick assessment of performance.

mAP serves as a composite indicator of model performance across multiple categories or various recall thresholds, commonly employed in target detection tasks. mAP is calculated by first determining the precision at different levels of recall for each category, followed by averaging these values across all categories. The F1 score, the harmonic mean of precision and recall, serves as a balanced metric between the two. It is particularly useful when both precision and recall need to be considered simultaneously. The formula for the F1 score is

(22) $$F1\;\mathrm{score}=2\times \frac{\mathrm{Precision}\times \mathrm{Recall}}{\mathrm{Precision}+\mathrm{Recall}}$$

The integrated application of these metrics enabled a multidimensional evaluation of the weed detection model’s performance, ensuring not only good performance on specific datasets but also effective identification capabilities in real agricultural environments.

## 4. Results and Discussion

### 4.1. Weed Recognition Experimental Results

This experiment was designed to evaluate the performance of different models in the task of weed recognition, thereby identifying the most effective algorithm to improve the accuracy of agricultural image analysis. We ccompared multiple models, including DETR, YOLOv9, EfficientDet, TinySegformer, and the proposed model. To further validate the advantages of the proposed model in practical applications, we conducted a comparative experiment with lightweight mobile models such as MobileNetV1 and MobileNetV3. The experiment focused on several key performance metrics: precision, recall, accuracy, mAP, and F1 score. These metrics comprehensively reflect the models’ abilities in weed recognition, helping researchers gain insights into the strengths and weaknesses of each model, thus providing a basis for further optimization. The experimental results are shown in Table 2.

In the experimental results, the DETR model achieved a precision of 0.83, a recall of 0.80, an accuracy of 0.81, a mAP of 0.81, and an F1 score of 0.81. This indicated that although DETR had certain advantages in handling object detection tasks, its ability to capture details and maintain stability in actual recognition required improvement. The YOLOv9 model performed slightly better than DETR, with a precision of 0.85, a recall of 0.82, an accuracy of 0.83, a mAP of 0.82, and an F1 score of 0.83. This result showed that YOLOv9 maintained a good balance in object detection, making it suitable for real-time detection scenarios. Next, EfficientDet produced further enhanced performance, achieving a precision of 0.88, a recall of 0.85, an accuracy of 0.87, a mAP of 0.86, and an F1 score of 0.86. This result reflected the superiority of EfficientDet in model efficiency and detection capabilities, especially in dealing with small objects and complex backgrounds. TinySegformer excelled in all metrics, achieving a precision of 0.90, a recall of 0.87, an accuracy of 0.89, a mAP of 0.88, and an F1 score of 0.88, demonstrating its advantages in fine-grained feature extraction and complex image segmentation. Finally, the proposed model achieved the highest precision of 0.92, recall of 0.89, accuracy of 0.91, mAP of 0.91, and F1 score of 0.90, significantly outperforming the other models, indicating its strong ability to process agricultural weed images. The experimental results from the different models reflect their characteristics in feature extraction and high-dimensional data processing. The DETR model utilizes a self-attention mechanism, which performs well in capturing long-range dependencies but shows relative weakness in handling details within complex images, thus limiting its performance. We added two lightweight models, MobileNetV1 and MobileNetV3, which are specifically optimized for mobile devices. The MobileNetV1 model achieved a precision of 0.71, a recall of 0.69, an accuracy of 0.71, mAP of 0.71, and F1 score of 0.70, while MobileNetV3 achieved a precision of 0.75, a recall of 0.77, an accuracy of 0.76, a mAP of 0.73, and an F1 score of 0.76. These models were included to provide a comparison of how lightweight neural networks perform in a mobile application context, particularly in terms of computational efficiency and recognition ability. The experimental results showed that the proposed model outperformed the other models, including the lightweight models. It achieved the highest precision of 0.92, recall of 0.89, accuracy of 0.91, mAP of 0.91, and F1 score of 0.90. This indicates the proposed method’s strong capability in handling agricultural weed images, especially when dealing with complex and varied environmental conditions.

When compared to the lightweight models, both MobileNetV1 and MobileNetV3 provided solid performance in terms of precision and recall, but they still fell short in comparison to the proposed method. The proposed model’s architecture, which integrates latent-based diffusion and transformer mechanisms, gives it a distinct advantage in feature extraction and context understanding. These strengths result in higher accuracy and F1 scores, which are crucial for practical weed detection applications in agricultural settings. In contrast, YOLOv9 provided a more balanced recognition ability through an improved convolutional neural network structure and multiscale feature fusion, making it suitable for real-time detection scenarios. EfficientDet maintained good recognition performance even in resource-constrained environments thanks to its design, which incorporates adaptive resource allocation and efficient feature extraction strategies. TinySegformer optimizes the image processing workflow by combining multimodal features and a lightweight design, particularly excelling in the processing fine-grained features. The proposed model integrates these advantages further, effectively enhancing feature expression and model robustness through a specific network architecture and the design of the latent loss function, ultimately achieving the best recognition results.

From a mathematical perspective, the performance of models is often closely related to the designs of their loss functions. The latent loss function adjusts the importance of the features, allowing the model to focus more on the key features that influence recognition results during training. Additionally, the effective adjustment of gradients and the rational fusion of features during the optimization process lead to more representative final output results, further improving accuracy and stability. Therefore, the experimental results not only reflect the characteristics of each model but also reveal directions that need to be emphasized when designing and optimizing models, providing valuable guidance for subsequent research.

### 4.2. Confidence Interval Analysis for Model Performance

To ensure the robustness and stability of the proposed method, we conducted 10 repeated experiments and calculated the standard deviation for each of the evaluation metrics (precision, recall, accuracy, mAP, and F1 score), as shown in Table 3. Based on the standard deviations, we further computed the confidence intervals for these metrics. Confidence intervals provide an estimate of the range within which the true values of the metrics lie, offering a measure of the variability across multiple repetitions. The results are summarized in the table below: the confidence intervals were computed at a 95% confidence level.

The standard deviations were calculated for each of the evaluation metrics across the 10 repetitions. Using these standard deviations, we computed the confidence intervals for each metric as follows:

(23) $$\mathrm{Confidence}\;\mathrm{Interval}=\mathrm{Metric}\;\mathrm{Value}\pm 1.96\times \frac{\sigma }{\sqrt{n}}$$

where σ is the standard deviation of the metric, and *n* is the number of repetitions (in this case, 10). The factor 1.96 corresponds to a 95% confidence level for the confidence interval.

The results show that the confidence intervals for the proposed method are narrow, indicating consistent performance across repetitions. This further confirms the robustness of the proposed method in the weed detection task. By computing these confidence intervals, we can confidently assert that the proposed model provides reliable and stable performance, making it suitable for practical deployment in real-world agricultural environments.

### 4.3. Ablation Experiment of Different Diffusion Subnetworks

The purpose of the experimental design was to assess the impact of various diffusion subnetworks on the task of weed detection, particularly comparing the performance of the residual diffusion network, diffusion transformer, and latent-based diffusion subnetwork in feature extraction and image understanding. This ablation experiment helped identify the optimal network architecture, providing data support for subsequent model optimization and practical applications.

From the experimental results presented in Table 4, it can be seen that the residual diffusion network achieved a precision of 0.75, a recall of 0.71, an accuracy of 0.73, a mAP of 0.72, and an F1 score of 0.73. This indicates that the network has limited capability in feature extraction and reconstruction. Although it employs a residual structure to enhance the learning of deep features, its understanding of complex images still falls short. Next, the performance of the diffusion transformer showed slight improvement, with a precision of 0.81, a recall of 0.78, an accuracy of 0.80, a mAP of 0.81, and an F1 score of 0.79. This model has an enhanced ability to capture the important features in images by combining the diffusion mechanism with the self-attention characteristics of transformers. However, compared to the latent-based diffusion subnetwork, the performance of the diffusion transformer remains insufficient. Finally, the latent-based diffusion subnetwork excelled in all metrics, achieving a precision of 0.92, a recall of 0.89, an accuracy of 0.91, a mAP of 0.91, and an F1 score of 0.90. This significant improvement is primarily due to its effective extraction and reconstruction of features in the latent space, fully utilizing image details and contextual information to enhance overall recognition accuracy and stability. Analyzing the different models provides insights into their mathematical characteristics and structural design. The residual diffusion network primarily relies on residual connections to mitigate the vanishing gradient problem in deep networks. While this can enhance the flow of information in some cases, the model struggles to effectively capture key details when processing complex images, thereby limiting its performance. The diffusion transformer improves feature representation through self-attention mechanisms, which can dynamically focus on important parts; however, its computational complexity when facing high-dimensional data may lead to performance bottlenecks. In contrast, the latent-based diffusion subnetwork introduces latent representations, allowing for processing image features at a higher level of abstraction. Mathematically, this representation provides the model with greater flexibility in capturing features, effectively reducing redundant information while retaining crucial data. During training, this model adjusts parameters appropriately, ensuring effective gradient propagation and avoiding overfitting, resulting in significant improvements across various performance metrics.

### 4.4. Ablation Experiment with Different Attention Mechanisms

In this section, the purpose of the experimental design was to evaluate the performance of various attention mechanisms in the task of weed detection, aiming to identify which mechanism can more effectively extract and enhance image features. By comparing the performance of the standard self-attention mechanism, convolutional block attention module, and latent attention mechanism, the experiment sought to explore how these attention mechanisms influence the overall effectiveness of the model, providing theoretical support for model design and optimization.

According to the experimental results shown in Table 5, the standard self-attention mechanism achieved a precision of 0.75, a recall of 0.72, an accuracy of 0.73, a mAP of 0.74, and an F1 score of 0.73. This indicates that although the standard self-attention mechanism has certain advantages in capturing the relationships between input features, its performance remains inadequate when handling complex image data. This may be due to limitations in dynamically adjusting the weights between diverse features, leading to the insufficient capture of image details. Next, the performance of the convolutional block attention module showed improvement, with a precision of 0.85, a recall of 0.81, an accuracy of 0.83, a mAP of 0.84, and an F1 score of 0.83. This module enhances the focus on key features during the feature extraction process by combining convolutional features with attention mechanisms, resulting in better performance when processing complex images. However, compared to the latent attention mechanism, the convolutional block attention module still exhibits limitations in feature fusion and representation capabilities. Finally, the latent attention mechanism demonstrates the best performance, with a precision of 0.92, a recall of 0.89, an accuracy of 0.91, a mAP of 0.91, and an F1 score of 0.90. This result indicates that the latent attention mechanism is more effective in weighting the importance of features, enabling the model to more accurately identify key features and enhance overall detection accuracy and stability. The experimental results of the different attention mechanisms reflect their characteristics in feature extraction and information integration. The advantage of the standard self-attention mechanism lies in its ability to dynamically adjust information flow by calculating relationships between input features, which allows the model to perform well with sequential data. However, in high-dimensional image data, this mechanism may fail to effectively capture multilevel features, leading to limited accuracy in recognizing complex images. The convolutional block attention module improves the richness of feature representation by combining the outputs of convolutional layers with attention mechanisms, partially overcoming the shortcomings of the standard self-attention mechanism. However, the limitation of this module lies in its reliance on fixed convolutional kernels for feature extraction and selection, which may not flexibly adapt to varying image contents. In contrast, the latent attention mechanism introduces latent space, making the feature extraction process more flexible. Mathematically, the latent attention mechanism utilizes the feature distribution in the latent space to select important features in a weighted manner. This mechanism enables greater flexibility in feature selection and fusion, ensuring that significant features are highlighted during the model training process. Thus, the differences in experimental results not only reflect the characteristics of different attention mechanisms in structural design but also reveal their strengths and weaknesses in feature capture and processing capabilities.

### 4.5. Lightweight Model Deployment

In modern agricultural applications, the lightweight design and deployment of models on mobile devices are particularly important. This paper developed a lightweight model capable of running on mobile terminals (such as the iPhone 13, (Apple, Cupertino, CA, USA)) to achieve efficient weed detection. The development process of this deployment encompassed multiple stages, including model design, optimization, compression, and final implementation on mobile platforms. First, during the model design phase, the architecture based on latent diffusion transformer was selected as the foundational model. This model has demonstrated superior performance in analyzing complex agricultural images through latent space feature extraction and self-attention mechanisms. However, the standard model often poses challenges for direct application on mobile devices due to its numerous parameters and computational complexity. Therefore, the introduction of lightweight techniques became crucial. In the optimization process, techniques such as pruning and quantization were employed. Pruning reduces the model’s size and computational demands by removing redundant parameters. Mathematically, the impact of pruning can be expressed as follows:

(24) ReducedModelSize=OriginalModelSize×(1−PruningRatio)

where the pruning ratio indicates the proportion of parameters removed. By selectively pruning parameters that do not affect model performance, the storage and computational footprint of the model can be effectively minimized. During quantization, floating-point parameters are converted to low-precision formats (such as INT8) to reduce memory usage and accelerate inference speed. The basic process of quantization can be represented as

(25) $$\mathrm{Quantized}\;\mathrm{Weight}=\mathrm{Round}\left(\frac{W-W_{\min}}{W_{\max}-W_{\min}}\times \left(2^{n}-1\right)\right)$$

where *W* represents the original weights, $W_{\min}$ and $W_{\max}$ are the minimum and maximum values of the weights, respectively, and *n* is the number of quantization bits. Quantization significantly reduces the computational load of the model while maintaining relatively high accuracy. After applying these lightweight techniques, transfer learning and fine tuning were conducted to ensure that the model retained good performance after deployment on mobile devices. During this process, data augmentation techniques were employed to further enhance the model’s robustness, expanding the training set and adjusting training strategies to improve the model’s adaptability in new environments. Finally, following the optimizations and adjustments, the lightweight model was successfully deployed on an iPhone 13. By using frameworks such as Core ML, the trained model could be converted into a format suitable for iOS platforms, enabling real-time weed detection. In practical implementation, mobile GPU acceleration technologies are utilized to ensure that high recognition accuracy is maintained while optimizing inference speed, allowing the model to respond quickly in real-world applications.

### 4.6. Limitations and Future Work

In this study, although an efficient weed detection model based on latent diffusion transformer was developed and successfully deployed in a lightweight manner, there are still several limitations and directions for future improvement. First, while the current model exhibits outstanding capabilities in feature extraction and understanding, it may still experience some recognition errors when faced with complex scenarios and varying environments. This is primarily due to the model’s reliance on a specific training dataset during the training process; when the conditions in real-world applications differ significantly from those during training, the model’s performance may be affected. Therefore, future research should focus on enhancing the model’s generalization ability by utilizing larger and more diverse datasets, which would include a wider range of environmental conditions, crop types, and lighting variations to improve its applicability in different agricultural scenarios. Secondly, although lightweight techniques were introduced to adapt the model to mobile terminals, the recognition accuracy of the model still requires improvement in extreme conditions, such as low light or highly disruptive backgrounds. To address these issues, further research could explore additional image enhancement and preprocessing techniques, such as advanced denoising algorithms or data augmentation methods like contrast adjustment and exposure correction, which could boost the model’s robustness under various lighting and background conditions. Additionally, for multitarget detection tasks in complex backgrounds, the model may require stronger separation capabilities, and this area of optimization, especially for crowded scenes with overlapping weeds, deserves deeper exploration.

From a practical standpoint, the results of this model can be integrated into real-world agriculture by helping farmers make more timely and precise decisions regarding herbicide use. By identifying the presence and type of weeds in real time, the model can enable farmers to apply herbicides only where necessary, thus reducing the overall amount of chemicals used in agricultural fields. This approach helps to not only to decrease the environmental impact of excessive chemical use but also contributes to more sustainable farming practices. Additionally, reducing chemical application can lower production costs and improve crop yield by preventing the overuse of herbicides, which can damage crops. The model can also be integrated with existing agricultural technologies, such as drones and mobile applications, for real-time weed monitoring. By coupling the weed detection model with drone systems, farmers can obtain aerial views of their fields, enabling high-efficiency monitoring over large areas. Drones can capture images of the crops, which are then processed by the model to identify weed patches, offering real-time feedback for precise herbicide application. Moreover, integrating the model with mobile applications can provide farmers with on-site analysis, allowing them to receive instant notifications about weed conditions on their smartphones and make quick decisions without needing to rely on external experts.

However, the adoption of this technology may be faced with several barriers. First, the need for training is a significant challenge, as many farmers may not be familiar with using such advanced technologies. To address this, user-friendly mobile applications, training programs, and support services should be developed to assist farmers in understanding how to use the technology effectively. Second, the cost of mobile devices equipped with the necessary features, such as high-quality cameras and sufficient processing power, may be prohibitive for some farmers, especially in resource-limited regions. To overcome this barrier, affordable and accessible mobile devices could be promoted, or alternative solutions like low-cost sensors could be used to complement this technology. Additionally, collaboration with governmental or agricultural organizations to provide subsidies or incentives for purchasing such devices could help increase adoption. Finally, the issue of model interpretability has not yet been fully addressed. While the model performs well in recognizing weeds, its decision-making process lacks transparency, which could raise trust issues in practical applications. Therefore, future research could aim to develop interpretable models that allow users to understand how the model makes specific decisions. By providing explanations for model predictions, farmers will be able to trust the model’s outputs and use them more effectively in the field. This would help enhance user trust and promote the widespread application of the technology.

## 5. Conclusions

In modern agriculture, the management and detection of weeds are crucial for the healthy growth of crops and the increase in yield. With advancements in technology, DL techniques have provided new solutions for agricultural image analysis. This study developed an efficient weed detection method based on the latent diffusion transformer model, aiming to enhance recognition accuracy and applicability. Through the DL analysis of agricultural images, the real-time monitoring of weeds was achieved, providing farmers with scientific evidence to optimize crop management. The innovation of this study lies in the combination of latent space feature extraction techniques and self-attention mechanisms to construct a lightweight model suitable for mobile devices. The model’s structural design was optimized, employing a latent loss function that effectively improves performance in handling complex agricultural images by weighting the importance of features. Additionally, lightweight techniques such as pruning and quantization were utilized, allowing the model to respond quickly on mobile devices and meet practical application needs. In experiments, the proposed model was validated against several mainstream models, demonstrating its superiority. The experimental results showed that the proposed model achieved a precision of 0.92, a recall of 0.89, an accuracy of 0.91, a mAP of 0.91, and an F1 score of 0.90. These metrics reflect the model’s strong capabilities in weed detection tasks, indicating its effectiveness in dealing with image analysis in complex scenarios. Furthermore, ablation experiments revealed that the latent-space-based diffusion subnetwork outperformed traditional models in feature extraction, achieving significant improvements in detection accuracy and stability. This research not only provides a new perspective for agricultural image analysis but also demonstrates the immense potential of DL technologies in practical applications. By integrating advanced model architectures and lightweight technologies, the findings of this study provide robust support for future agricultural management and decision making, driving the development of smart agriculture. Future work can focus on further expanding the dataset to capture a wider variety of weed species and environmental conditions, including different lighting and weather conditions, to improve the model’s generalization ability. Moreover, exploring more advanced or hybrid modeling architectures that combine the strengths of computer vision with other AI methods, such as reinforcement learning or generative models, could enhance the model’s robustness and adaptability in real-world scenarios. Additionally, conducting field trials in collaboration with farmers or agricultural organizations would allow for the evaluation of the model’s performance in real agricultural contexts, helping to refine its application in practical, real-time weed management.

## Figures and Tables

**Table 1:** Dataset distribution.

Species	Quantity
*Amaranthus retroflexus*	1679
*Xanthium spinosum*	904
*Xanthium sibiricum*	1230
*Amaranthus palmeri*	1493
*Xanthium italicum*	1551
*Amaranthus hybridus*	828

**Table 2:** Weed recognition experiment results.

Model	Precision	Recall	Accuracy	mAP	F1 Score
DETR	0.83	0.80	0.81	0.81	0.81
YOLOv9	0.85	0.82	0.83	0.82	0.83
EfficientDet	0.88	0.85	0.87	0.86	0.86
TinySegformer	0.90	0.87	0.89	0.88	0.88
MobileNetV1	0.71	0.69	0.71	0.71	0.70
MobileNetV3	0.75	0.77	0.76	0.73	0.76
Proposed Method	0.92	0.89	0.91	0.91	0.90

**Table 3:** Confidence intervals for weed recognition metrics (10 repetitions).

Model	Precision	Recall	Accuracy	mAP	F1 Score
DETR	0.83±0.03	0.80±0.04	0.81±0.03	0.81±0.02	0.81±0.03
YOLOv9	0.85±0.02	0.82±0.03	0.83±0.02	0.82±0.02	0.83±0.02
EfficientDet	0.88±0.02	0.85±0.03	0.87±0.02	0.86±0.02	0.86±0.02
TinySegformer	0.90±0.02	0.87±0.03	0.89±0.02	0.88±0.02	0.88±0.02
Proposed Method	0.92±0.02	0.89±0.03	0.91±0.02	0.91±0.02	0.90±0.02

**Table 4:** Ablation Experiment with different diffusion subnetworks.

Diffusion	Precision	Recall	Accuracy	mAP	F1 Score
Residual Diffusion Network	0.75	0.71	0.73	0.72	0.73
Diffusion Transformer	0.81	0.78	0.80	0.81	0.79
Latent-Based Diffusion Subnetwork	0.92	0.89	0.91	0.91	0.90

**Table 5:** Ablation Experiment with different attention mechanisms.

Diffusion	Precision	Recall	Accuracy	mAP	F1 Score
Standard Self-Attention	0.75	0.72	0.73	0.74	0.73
Convolutional Block Attention Module	0.85	0.81	0.83	0.84	0.83
Latent Attention	0.92	0.89	0.91	0.91	0.90

## Data Availability

The data presented in this study are available upon request from the corresponding author.
